# Therapeutic hypothermia after cardiac arrest: outcome predictors

**DOI:** 10.5935/0103-507X.20150056

**Published:** 2015

**Authors:** Rodrigo Nazário Leão, Paulo Ávila, Raquel Cavaco, Nuno Germano, Luís Bento

**Affiliations:** 1 Unidade de Urgência Médica, Centro Hospitalar de Lisboa Central - EPE - Lisboa, Portugal.

**Keywords:** Hypothermia, induced, Heart arrest, Cardiopulmonary resuscitation, Neuron-specific enolase, Hypoxia-ischemia, brain

## Abstract

**Objective:**

The determination of coma patient prognosis after cardiac arrest has clinical, ethical and social implications. Neurological examination, imaging and biochemical markers are helpful tools accepted as reliable in predicting recovery. With the advent of therapeutic hypothermia, these data need to be reconfirmed. In this study, we attempted to determine the validity of different markers, which can be used in the detection of patients with poor prognosis under hypothermia.

**Methods:**

Data from adult patients admitted to our intensive care unit for a hypothermia protocol after cardiac arrest were recorded prospectively to generate a descriptive and analytical study analyzing the relationship between clinical, neurophysiological, imaging and biochemical parameters with 6-month outcomes defined according to the Cerebral Performance Categories scale (good 1-2, poor 3-5). Neuron-specific enolase was collected at 72 hours. Imaging and neurophysiologic exams were carried out in the 24 hours after the rewarming period.

**Results:**

Sixty-seven patients were included in the study, of which 12 had good neurological outcomes. Ventricular fibrillation and electroencephalographic theta activity were associated with increased likelihood of survival and improved neurological outcomes. Patients who had more rapid cooling (mean time of 163 versus 312 minutes), hypoxic-ischemic brain injury on magnetic resonance imaging or neuron-specific enolase > 58ng/mL had poor neurological outcomes (p < 0.05).

**Conclusion:**

Hypoxic-ischemic brain injury on magnetic resonance imaging and neuron-specific enolase were strong predictors of poor neurological outcomes. Although there is the belief that early achievement of target temperature improves neurological prognoses, in our study, there were increased mortality and worse neurological outcomes with earlier target-temperature achievement.

## Abstract (pt)

**Objetivo:**

A determinação do prognóstico de pacientes em coma após parada cardíaca tem implicações clínicas, éticas e sociais. Exame neurológico, marcadores de imagem e bioquímicos são ferramentas úteis e bem aceitas na previsão da recuperação. Com o advento da hipotermia terapêutica, tais informações devem de ser confirmadas. Neste estudo procurou-se determinar a validade de diferentes marcadores que podem ser utilizados na detecção de pacientes com mau prognóstico durante um protocolo de hipotermia.

**Métodos:**

Foram coletados prospectivamente os dados de pacientes adultos, internados após parada cardíaca em nossa unidade de terapia intensiva para realização de protocolo de hipotermia. Nosso intuito foi realizar um estudo descritivo e analítico para analisar a relação entre os dados clínicos, parâmetros neurofisiológicos, de imagem e bioquímicos, e o desfecho após 6 meses, conforme definido pela escala *Cerebral Performance Categories* (bom, se 1-2, e mau, se 3-5). Foi coletada uma amostra para determinação de neuroenolase após 72 horas. Os exames de imagem e neurofisiológicos foram realizados 24 horas após o período de reaquecimento.

**Resultados:**

Foram incluídos 67 pacientes, dos quais 12 tiveram evolução neurológica favorável. Fibrilação ventricular e atividade teta no eletroencefalograma se associaram a bom prognóstico. Pacientes submetidos a resfriamento mais rápido (tempo médio de 163 *versus* 312 minutos), com lesão cerebral causada por hipóxia/isquemia detectada na ressonância nuclear magnética ou níveis de neuroenolase superiores a 58ng/mL se associaram a desfecho neurológico desfavorável (p < 0,05).

**Conclusão:**

A presença de lesão cerebral causada por hipóxia/isquemia e de neuroenolase foram fortes preditores de má evolução neurológica. Apesar da crença de que atingir rapidamente a temperatura alvo da hipotermia melhora o prognóstico neurológico, nosso estudo demonstrou que este fator se associou a um aumento da mortalidade e a uma pior evolução neurológica.

## INTRODUCTION

Therapeutic hypothermia consists of the controlled reduction of patient core temperature with predefined therapeutic goals. Hypothermia is defined as mild (32 - 34°C), moderate (28 - 32°C) or deep (< 28°C).^(1)^ There is evidence of the use of therapeutic hypothermia since the ancient Egyptian, Greek and Roman civilizations.^(2)^ It was used to treat pain, induction of "cerebral silence" during surgery, tetanus, traumatic brain injury or even epilepsy.^(3)^ In the 1930s and 1940s, studies by Temple Fay, Claude Beck and Charles Bailey in patients with head trauma and cardiac surgery, demonstrated its neuroprotective capacity.^(4,5)^ In the 1990s, it was demonstrated that hypothermia for 2 to 24 hours in patients with traumatic brain injury, stroke and cardiac arrest significantly improved their neurological recovery.^(6-8)^ Currently, the main applications are neurological protection after cardiopulmonary resuscitation (CPR -with a target temperature of 33 ± 1° C), during cardiac or neurological highly complex surgery, in the treatment of refractory intracranial hypertension, as well as in some diseases that occur with increasing temperature.^(9)^

Recently Nielsen et al. published a trial comparing two target temperatures (33°C and 36°C), which showed no advantage of hypothermia in terms of mortality and neurologic outcomes at 6 months.^(10)^ Despite these findings, one cannot draw definite conclusions, and these results must be applied in other populations. Therefore, International Liaison Committee on Resuscitation (ILCOR) published a statement recommending the use of 2010 guidelines.^(11,12)^

Post-cardiac arrest results are predominantly dependent on neurological recovery after a period of anoxia. The prediction of neurologic outcomes is important to decide the extent of measures or limits of post-arrest care.^(13)^ In 2006, the American Academy of Neurology (AAN) published guidelines for the establishment of poor prognosis patients in comatose survivors of CPR. The absence of pupillary or corneal reflexes and no response or response in extension at 72 hours after arrest, the absence of N20 somatosensory evoked potentials, neuron specific enolase (NSE) levels higher than 33ng/mL at 72 hours and presence of myoclonic status epilepticus in the first 24 hours were considered poor prognostic factors.^(14)^ However, these guidelines were mainly based on studies of patients not undergoing therapeutic hypothermia, and there are new studies showing that AAN guidelines may not apply for those patients submitted to therapeutic hypothermia.^(15,16)^ Recent evidence suggests that it may be important to establish the prognosis for patients undergoing therapeutic hypothermia: brainstem reflexes,^(17,18)^ motor response,^(19)^ myoclonus,^(20,21)^ electrophysiology,^(22-28)^ biomarkers^(17,29-32)^ and magnetic resonance imaging (MRI).^(17,33-35)^ Despite several studies, univariate analyses have not been sufficiently robust or consistent to arrive at one prognostic factor that can safely predict the prognosis. Currently, the recommended best strategy is to deduce the prognosis by an integrated assessment of predictors and all clinical information. Therefore, we conducted this study to determine the validity of different markers, which can be used in the detection of patients with poor prognosis under therapeutic hypothermia.

## METHODS

We conducted a prospective study between May 2012 and June 2014 in an intensive care unit (ICU) at the *Hospital de São José*, Central Lisbon Hospital Center, Portugal, to determine outcome predictors in patients after cardiac arrest who were admitted and subjected to a hypothermia protocol. The study was approved by the Ethics Committee of Centro Hospitalar de Lisboa Central - EPE, in accordance with the Declaration of Helsinki, approval letter 253/2015. Informed consent was dispensed.

### Hypothermia protocol

Our hypothermia protocol was designed based on recommendations published by ILCOR in 2010 and includes the emergency medical services (EMS), the emergency department and the coronary care unit.^(11)^

**Figure 1:**
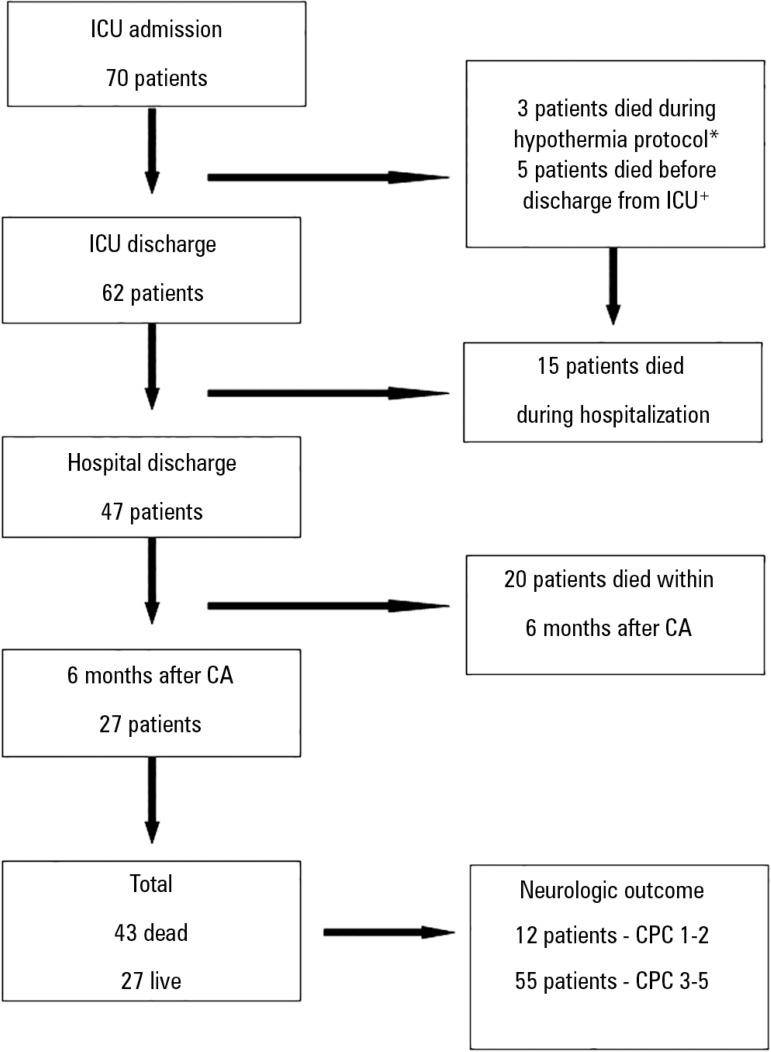
Mortality and neurologic outcomes. ICU - intensive care unit; CA - cardiac arrest; CPC - Glasgow-Pittsburgh cerebral performance categories. * These patients were excluded for not having completed the hypothermia protocol. ^+^ These patients died between the 5^th^ and 11^th^ days after cardiac arrest.

**Figure 2:**
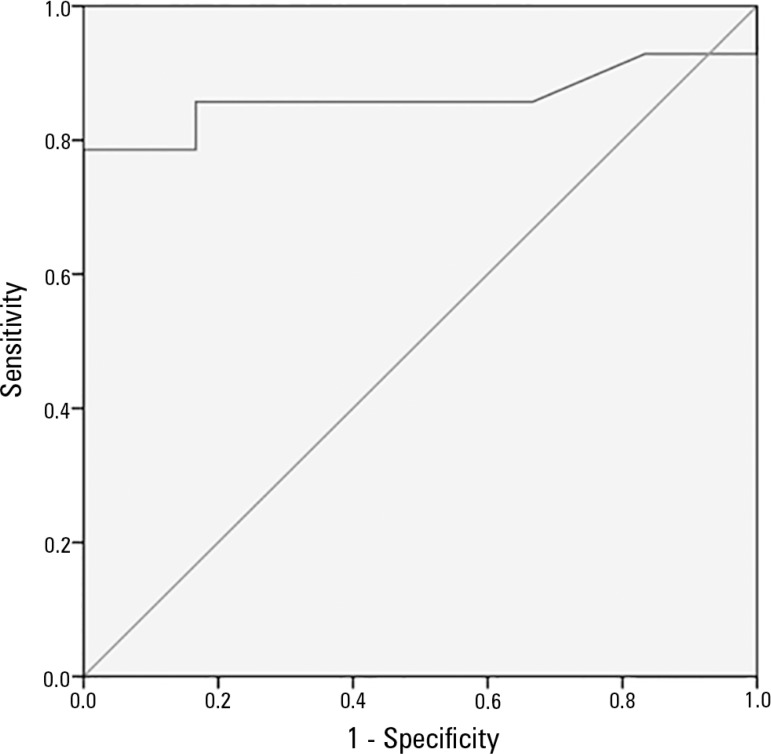
Neuron specific enolase ROC curve.

All patients with maintained systolic blood pressure superior to 80mmHg after return of spontaneous circulation and Glasgow coma score lower (GCS) than 9 were included. Patients with a core temperature lower than 30°C, coagulopathy, cryoglobulinemia, severe bleeding, intracerebral hemorrhage and known terminal illness were excluded. Figures 1S and 2S of electronic supplementary materials detail the methods for inducing hypothermia. After achieving the target temperature (33 ± 1°C), hypothermia was maintained during 24 hours using cooling blankets (Blanketrol^®^ III, Cincinnati Sub-Zero) in automatic mode. During this period, it was provided if necessary: hemodynamic support for mean arterial pressure > 80mmHg; ventilatory support for normal ventilation; renal support for diuresis > 1mL/kg/h; continuous enteral nutrition, if tolerated, from the beginning with a rate < 20mL/h; blood glucose control for levels below 200mg/dL; ion control (K^+^ > 4mEq/L and Mg^2+^ > 2.5mEq/L). For instances of refractory hypotension, refractory arrhythmias or uncontrollable bleeding, the protocol was discontinued. Rewarming was performed up to 36°C (with maximum temperature rise of 0.3°C/hour, over a minimum period of 8 hours) only with the superior blanket. During this period, the following tasks were performed: the hourly monitoring of blood glucose, suspension of curarization when temperature > 36°C, suspension of sedation when temperature > 36°C and Train of Four 4/4, suspension of K^+^ replacement six hours before rewarming unless serum levels below 3mEq/L, suspension of Mg^2+^ replacement when temperature > 36°C, use of antipyretics to avoid temperatures > 37°C, and temperature maintenance < 37°C during the subsequent 72 hours.

We analyzed the following demographic and clinical data: sex, age, Simplified Acute Physiology Score II^(36)^ and 3 (SAPS II and SAPS 3) and Acute Physiology and Chronic Health Evaluation II (APACHE II).^(37)^ We characterized cardiac arrest accordingly with its causes, place of occurrence and initial rhythm. Hypothermia protocol times (time until CPR, CPR duration, time to return of spontaneous circulation (ROSC), time from ROSC until the beginning of hypothermia, time until achieving target temperature, hypothermia duration, and rewarming duration) were registered. Seventy-two hours after cardiac arrest, the motor responses, pupillary responses and corneal reflexes were evaluated, and the GCS was calculated. We also included lactate evaluation at admission, 24 hours and its variation, NSE at 72 hours, electroencephalogram (EEG) results, N20 somatosensory evoked potentials and signs of anoxic encephalopathy (hypoxic-ischemic brain injury) at MRI, all made in the 24 hours after the rewarming period. For statistical analysis, we considered hypoxic-ischemic brain injury as the presence (in MRI) of abnormalities in large areas, such as the cerebellum, cerebral cortex and basal ganglia, in diffusion-weighted imaging, apparent diffusion coefficient map and fluid-attenuated inversion recovery sequences.

The neurologic outcome at 6 months was determined by in-person consultation or by phone using the Glasgow-Pittsburgh cerebral performance categories scale (CPC). This scale is divided into five categories: 1) conscious and alert with normal function or only slight disability, 2) conscious and alert with moderate disability, 3) conscious with severe disability, 4) comatose or persistent vegetative state, 5) brain death or death from other causes.^(38)^ A CPC score of 1-2 was considered a good prognosis and 3-5 as a poor prognosis.

### Statistical analysis

In addition to the descriptive analysis, non-normally distributed continuous variables were analyzed with a Mann-Whitney U test, and Pearson's chi-squared was utilized to test categorical variables. A p < 0.05 was regarded as statistically significant. Receiver operating characteristic analysis (ROC) was performed to define a cut-off value with 100% of specificity for NSE.

To assess the significance of the variables on the probability of having a poor neurological prognosis, we performed multivariate analysis using logistic regression by the Forward:LR method. The Hosmer-Lemeshow test was used to verify the goodness of fit of the logistic regression model and ROC analysis was performed to define the discriminating capacity of the model.

Statistical analysis was performed with IBM Statistical Package for Social Science (SPSS) version 21 (International Business Machines Corp., USA).

## RESULTS

During the study period 70 patients were admitted and 67 concluded the hypothermia protocol. 27% were female (n = 18). Sixty-nine percent of the cardiac arrests occurred out of the hospital. The main causes of cardiac arrest were acute myocardial infarction (26 patients) and respiratory failure (18 patients). Mortality rate at 6 months was 61%. Twelve patients had good neurological outcomes (Table 1). Figure 1 shows the selection and evolution of patients over the 6-month follow-up.

**Table 1:** Characteristics of the study population.

Characteristics	Total
	(N = 67)
Age (year)	62.6 (13)
Gender, male	49 (73)
Location of cardiac arrest	
Out-of-hospital	46 (69)
Hospital	21 (31)
Causes of cardiac arrest	
Respiratory failure	18 (27)
Acute myocardial infarction	26 (39)
Dysrhythmia	3 (4)
Stroke	1 (2)
Metabolic disorders	2 (3)
Undetermined	17 (25)
Initial rhythm	
Ventricular tachycardia	1 (2)
Ventricular fibrillation	27 (40)
Asystole	27 (40)
Pulseless electrical activity	10 (15)
Undetermined	2 (3)
Place of hypothermia protocol beginning	
Emergency medical services	10 (15)
Emergency department	12 (18)
Intensive care unit	45 (67)
Mortality at 6 months	41 (61)
Good neurologic outcome (CPC 1-2)	12 (18)

CPC - Glasgow-Pittsburgh Cerebral Performance Categories; SD - standard deviation. Results expressed as a number (%) and mean (standard deviation).

The mean age was 62.6 ± 13 years, and this variable was not associated with neurologic outcome (p = 0.46). Our study group had at admission an average SAPS II of 63.4 ± 12.1, SAPS 3 of 76.5 ± 13.0 and APACHE II of 27.6 ± 7.0. Analyzing the severity scores, we found a significant relationship between high values of SAPS 3 and APACHE II with mortality at 6 months. However, there was no association between the severity scores and the neurological outcome (Table 2).

**Table 2:** Variables and neurologic outcomes at 6 months

	CPC 1-2	CPC 3-5	p value
	(N = 12)	(N = 55)	
Age (years)	59.5 (13.3)	63.0 (13.6)	0.46
Severity Scores			
APACHE II	25.5 (7.9)	28.5 (5.7)	0.15
SAPS II	60.5 (13.4)	63.3 (12.4)	0.20
SAPS 3	72.8 (10.1)	76.7 (14.5)	0.42
Initial rhythm			
Ventricular tachycardia	0 (0)	1 (2)	0.61
Ventricular fibrillation	8 (12)	17 (25)	0.01
Asystole	4 (6)	22 (33)	0.33
Pulseless electrical activity	0 (0)	10 (15)	0.08
Place of hypothermia protocol beginning			
Emergency medical services	2 (3)	8 (12)	0.68
Emergency department	2 (3)	10 (15)	0.97
Intensive care unit	8 (12)	37 (55)	0.73
Hypothermia protocol times in mins			
CA to CPR time	2.27 (4.58)	5.13 (6.73)	0.19
CPR duration	19.18 (17.62)	21.13 (11.82)	0.67
CA to ROSC time	21.45 (18.18)	26.26 (15.75)	0.44
ROSC to hypothermia time	168.64 (184.73)	124.49 (96.79)	0.29
Time until target temperature	31 1.82 (192.24)	163.44 (121.90)	< 0.01
Hypothermia duration	1478.4 (76.8)	1470.60 (101.40)	0.82
Rewarming duration	660 (241.2)	726.00 (382.20)	0.59
Lactate			
Admission	4.02 (2.71)	5.77 (3.57)	0.15
24 hours	2.41 (1.87)	2.54 (1.59)	0.84
24 hours variation	-1.46 (2.96)	-3.18 (3.51)	0.11
Hypoxic-ischemic brain injury in MRI	1 (2)	33 (49)	< 0.01
Absent N20 response on SSEP	6 (9)	10 (15)	0.07
EEG			
Theta activity	9 (13)	5 (7)	0.01
Delta activity	2 (3)	4 (6)	0.23
Status epilepticus	0 (0)	1 (2)	0.57
Burst-suppression activity	1 (2)	12 (18)	0.27
Neuron specific enolase	23.33 (17.75)	103.36 (75.80)	0.02

CPC - Glasgow-Pittsburgh cerebral performance categories; APACHE II - Acute Physiology And Chronic Health Evaluation II; SAPS - Simplified Acute Physiology Score; CA - cardiac arrest; CPR - cardiopulmonary resuscitation; EEG - electroencephalogram; min - minutes; ROSC - return of spontaneous circulation; MRI - magnetic resonance imaging; SSEP - somatosensory evoked potentials. Results expressed as a number (%) and mean (standard deviation).

Twenty-five patients initially had ventricular fibrillation, 26 had asystole and 10 had pulseless electrical activity. All patients with pulseless electrical activity had a fatal outcome up to 6 months (p = 0.01). From the good prognosis group (n = 12), 8 patients had ventricular fibrillation as initial rhythm. Those with ventricular fibrillation were associated with increased likelihood of survival (p = 0.03) and improved neurological outcome (p = 0.01; OR 0.17 [CI 0.04 - 0.76]) when compared with patients who initially had other rhythms (Table 2).

Forty-five patients initiated the hypothermia protocol in ICU, 12 patients in the emergency department and 10 with EMS. There was no significant difference regarding mortality at 6 months and neurologic outcome when compared the different groups (Table 2). The protocol implementation times ranged: time until CPR between 0 and 30 minutes, CPR duration between 2 and 64 minutes, time to ROSC between 4 and 85 minutes, time from ROSC until beginning of hypothermia between 0 and 10 hours, time until achieving target temperature between 0 and 12 hours, hypothermia duration between 20 and 31 hours, and rewarming duration between 6 and 38 hours. Note that just one of the patients exceeded the 8 hours limit to initiate hypothermia. It was a patient with acute myocardial infarction and ventricular fibrillation as initial rhythm, which had good neurologic outcome. The delay was because this patient was previously submitted to coronary angioplasty. Analyzing these periods, we have found that shorter times to reaching the target temperature were associated with higher mortality at 6 months (p = 0.04) and worse neurologic outcomes (p < 0.01). None of the other periods of the protocol demonstrated statistically significant relationships with the outcomes under study (Table 2).

The motor responses, pupillary responses, corneal reflexes were evaluated 72 hours after cardiac arrest, and the GCS was calculated. Absent motor reflexes or extensor posturing did not predict poor outcomes. Six patients had absent pupillary response to light and absent corneal reflexes, and none regained consciousness. The presence of motor responses, pupillary responses and corneal reflexes did not correlate with neurologic prognosis at 6 months. The GCSs ranged between 3 and 15 and had no statistically significant association with prognosis (p = 0.13).

We analyzed lactate at admission and 24 hours later as well as its variation during this period. Statistical analysis showed that higher lactate values at admission and 24 hours were tendentiously associated with greater mortality and worse neurological prognosis at 6 months. However, we found no statistically significant differences (Table 2).

Nearly all patients with hypoxic-ischemic brain injury in MRI had poor neurologic outcomes (p < 0.01). The occurrence of poor neurological prognosis in patients with the presence of hypoxic-ischemic brain injury was 19 times higher than in those without lesions (OR 19.8 [CI 95% 1.7-229.6]) (Table 2). None of the patients lacking a N20 response had good neurological outcomes. However, these results were without statistical significance (p = 0.07) (Table 2). The majority of the patients with good neurologic outcomes had theta activity. Therefore, the presence of EEG theta activity was associated with improved neurological outcomes (p = 0.01; OR 0.11 [CI 95% 0.01-0.75]). Other EEG patterns were not statistically significant (Table 2).

Higher NSE values were associated with worse neurologic outcomes at 6 months (p = 0.02) (Table 2). Receiver operating characteristic analysis revealed that NSE > 58ng/mL had a sensitivity of 79% and a specificity of 100% to poor neurological outcomes. The area under the curve (AUC) was 0.86, p < 0.001 (Figure 2).

**Figure 3:**
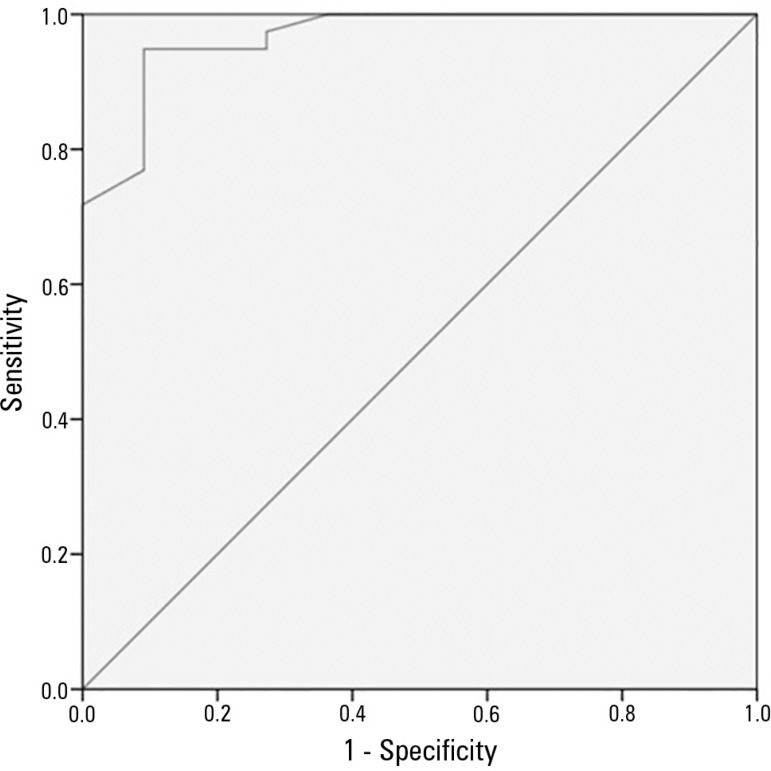
Adjusted model ROC curve.

In order to define the significance of these variables on the probability of having a poor neurological prognosis, we performed multivariate analysis using logistic regression by the Forward:LR method. This model generated the following significant variables: ventricular fibrillation (OR 0.013 [CI 95% 0.01 - 12:26], p = 0.05), time to reach target temperature (OR 0.98 [CI 95% 0.97 - 0.99], p = 0.02), presence of hypoxic-ischemic brain injury in MRI (OR 23.5 [CI 95% 2.7 - 204.6], p = 0.04), and NSE > 58ng/mL (OR 21.7 [CI 95% 12.3 - 56.5], p = 0.05). Receiver operating characteristic analysis revealed an excellent discriminating capacity with an AUC = 0.96, p < 0.001 (Figure 3). According this analysis, the probability of poor neurological prognosis decreases with the presence of ventricular fibrillation and increases with shortened time to reach the target temperature, presence of hypoxic-ischemic lesions on MRI and NSE values > 58ng/mL. The percentage of correct classifications according to this model is 94.7%.

## DISCUSSION

This study verified that several markers for the assessment of neurological outcome remain valid in patients submitted to therapeutic hypothermia after cardiac arrest. We found that ventricular fibrillation and EEG theta activity are protective factors, whereas shorter time to achieving target temperature, hypoxic-ischemic brain injury in MRI and NSE > 58ng/mL were associated with poor neurological outcomes. Absent pupillary response to light and absent corneal reflexes were associated with poor outcomes, which is consistent with the literature.^(16)^

We began by analyze the correlation between the severity scores at admission, mortality and neurologic outcome. Although it was found that the APACHE II and SAPS 3 are related to mortality at 6 months, the same is not true for neurological outcomes. These results are in accordance with recent findings that verified that the admission severity scores are not good predictors of neurologic outcomes. In fact, we determined that the predictive power of the APACHE II increases with time, and this score is only mildly correlated with the neurological outcome of those who had in-hospital cardiac arrest.^(39-41)^ We attribute this to the fact that important variables in this set, such as the cardiac arrest rhythm, the time of arrest and the time to onset of specialized CPR, were not included.^(42,43)^

Our study population consisted of 37 patients with non-shockable initial rhythm and 28 with shockable initial rhythm. We found that the presence of ventricular fibrillation was associated with a greater probability of favorable neurological outcomes, which is consistent with the published literature on patients undergoing therapeutic hypothermia.^(44,45)^

Findings in experimental studies suggested that the early initiation of therapeutic hypothermia after cardiac arrest would be a safe and beneficial treatment in terms of reduced mortality and improved neurological outcomes, which led to its institution in the prehospital environment.^(46)^ However, recent studies have failed to demonstrate these advantages.^(47-51)^ Our study confirms these findings, showing no improvement of prognosis when hypothermia was begun outside the hospital. In view of these findings, the reasons why we failed to show benefits are speculative. Early cooling was performed by trained personal and according to the established protocol. The study was not completely blind and, for this reason, we cannot exclude bias although we believe it is an unlikely explanation. Additionally, as this was an observational study, we cannot exclude the existence of known or even unknown yet confounding factors. Another possible explanation is the existence of side effects that may have contributed to this outcome. For example, hypothermia induced with infusion fluids is associated with decreased coronary perfusion pressure and decreasing pH and PaO_2_ which are related with poor outcomes.^(47,52)^ The early onset of cooling may be more beneficial if initiated during CPR, which, therefore, reduces reperfusion injury, which occurs after ROSC as demonstrated in animal studies.^(53)^ However, this assumption needs to be tested in future studies. As Nielsen et al. have suggested that the avoidance of hyperthermia can be as protective as hypothermia,^(10)^ we believe that the fact that we did not compared temperature level with mortality and neurologic outcome is a limitation of this study. This possibility was not considered because this study was designed and initiated before the publication of this study by Nielsen et al.. Furthermore, all patients fulfilled the hypothermia protocol and reached a target temperature of 33 ± 1°C. Just one of the patients exceeded the 8-hour limit to initiate hypothermia, which explains the 12-hour maximum for achieving the target temperature. The exclusion of this case from statistical analysis did not affect the final result.

In this study, we verified that a lower time to reach target temperature was associated with higher mortality and worse neurological outcomes at 6 months, which indicates that higher temperature reduction rates are associated with a worse prognosis. We hypothesize that this result may be related to the fact that patients with more severe or irreversible neurological damage are less reactive to low temperatures, so there is less shivering and less need for sedation allowing for faster cooling.^(54,55)^

Lactate is an important marker of hypoxia and/or hypoperfusion and its levels in serum and the effectiveness of its clearance are related to mortality in critically ill patients.^(56)^ This study demonstrated that higher lactate values and variations thereof at admission and 24 hours later were tendentiously associated to greater mortality and worse neurological prognosis at 6 months, although these findings were not statistically significant. Several studies have tried to verify the association of lactate measurements with mortality and neurologic prognosis, although the results are inconclusive. Some retrospective studies demonstrated an association of likely little clinical value between elevated lactate levels, mortality and worse neurological outcomes.^(57,58)^ A recent prospective study found a significant relationship between survival, good neurologic outcome, low serum values and high percentage of lactate clearance.^(59)^ We hypothesized that other variables such as short duration of cardiac arrest, efficiency and rapid onset of CPR, efficiency of fluid resuscitation or oxygenation efficiency are related with lower serum levels and higher lactate clearance and therefore probably with better prognosis. We believe that its use to predict outcome may be important in the future but for now there is not sufficient evidence to recommend it.

Of our patients with hypoxic-ischemic brain injury in MRI, only one had a good neurological outcome. This result is in accordance with other published studies, which indicate that the presence of extensive and severe ischemic brain injury in diffusion-weighted MRI is associated with poor neurological outcome providing additional prognosis information.^(33,60)^ Despite the good performance of MRI in our study, their interpretation is complex, is not fully standardized and is affected by significant inter-observer variability. Measurements in apparent diffusion coefficient appear to solve the problems of standardization and inter-observer variability, although its implementation requires software that is not universally available. Furthermore, it is an exam of limited access and is difficult in unstable patients. Considering these problems, the limited quality of the available literature and the small sample of our study, we do not recommend its use alone to define prognosis in post-cardiac arrest patients.^(33,61)^

Research on the use of somatosensory evoked potentials demonstrated that bilateral potential absence is a good predictor of poor neurologic outcome in comatose patients after cardiac arrest.^(62)^ Although in this study all the patients without a N20 response had poor neurologic outcomes, this finding was not statistically significant. We believe that the elevated number of patients with poor neurologic outcomes who had positive N20 responses influenced these results.

The univariate analysis of EEG patterns showed that theta activity was associated with better chance of good neurologic outcomes. Malign patterns were associated with poor neurologic outcomes, although these findings were not statistically significant. However, when performing multivariate analysis, EEG results were not included in the final equation. These results are in accordance with other published studies, which conclude that benign patterns are associated with a good prognosis.^(63,64)^ Although all patients were treated with sedative drugs during the period of hypothermia, when conducting EEG, 16 patients were still under sedation, all with fentanyl infusion (dose between 0.5 - 2.0µg/kg/h, which was suspended during the 24 hours after rewarming). Despite this, it is unlikely that the EEG patterns were altered by the use of fentanyl in the doses used.^(65,66)^ The precision of neurological outcome determinations by electroencephalography is limited by the lack of a cross-language regarding the malignant patterns. A universal and consensual classification directed to critically ill patients should be created to evidence its real importance on neurologic prognostic determination.^(67)^

Neuron-specific enolase is an enzyme that can be found in neuronal and neuroendocrine tissues, which has a 24-hour half-life. Because of its specificity for these tissues and the evidence that its value increases when there is neuronal destruction, it has gained importance as a prognostic marker in comatose patients after cardiac arrest. In 2006, the AAN^(68)^ defined a value higher than 33ng/mL between days 1-3 post-cardiac arrest as a predictor of poor neurologic outcome. However, this value was based mainly in studies with population that was not submitted to hypothermia.^(14)^ Since the implementation of this standard, several investigations have studied the possible effect of therapeutic hypothermia in this value with different results, indicating significant cut-off values from > 28 to > 97 ng/mL or even stating that therapeutic hypothermia does not affect its value.^(30,31,69,70)^ More recent studies have shown that not the absolute value but its variation can be more closely related with outcome.^(13,71)^ In our study, higher NSE values were associated with worse neurologic outcomes at 6 months having NSE > 58 ng/L a 100% specificity for poor prognosis, indicating that higher NSE values than those established by the AAN were associated with worse outcomes and, as such, that therapeutic hypothermia can in fact change the cut-off value of NSE for poor outcome.

Our study had some limitations. As data were collected by different observers, the study was subjected to inter-observer variability. The relative small study population may have influenced the results, with some of the traditional markers of poor neurological outcome as the absence of N20 response in somatosensory evoked potentials or presence of status epilepticus in EEG not having statistical significance. Although not included in the AAN recommendations, studies indicate that S-100β protein may be superior to NSE for determining the prognosis after cardiac arrest.^(32)^ However, its value is less well documented and more controversial than NSE.^(68)^ This biomarker has not been evaluated because it was not available in our laboratory when the study was designed, but it may have an important role in determining neurological outcomes.

A strength of this study is that it is based on a well-established protocol including an intensive coronary unit and EMS, equipped to initiate therapeutic hypothermia in out-of-hospital environments on a routine basis, without identified side effects.

## CONCLUSION

Hypoxic-ischemic brain injury on magnetic resonance imaging and neuron specific enolase were strong predictors of poor neurological outcomes. Although there is a belief that the early achievement of target temperature improves neurological prognosis, in our study there were increased mortality and worse neurological outcomes with earlier target-temperature achievement.

Despite the fact that these results have important and statistically significant value, these results need to be replicated in larger multicenter prospective randomized studies to generate consensus.
