# Supplementary material for: Therapeutic hypothermia after cardiac arrest: outcome predictors
Source: Rev Bras Ter Intensiva. 2015 Oct-Dec;27(4):322–32. doi: 10.5935/0103-507X.20150056 (PMC4738817; doi:10.5935/0103-507X.20150056)
Supplement: Supplementary file 1 [file rbti-27-04-0322-suppl01.pdf]

Therapeutic hypothermia after cardiac arrest: outcome predictors

Hipotermia terapêutica após parada cardíaca: preditores de prognóstico

Rodrigo Nazário Leão[1], Paulo Ávila[1], Raquel Cavaco[1], Nuno Germano[1], Luís Bento[1]

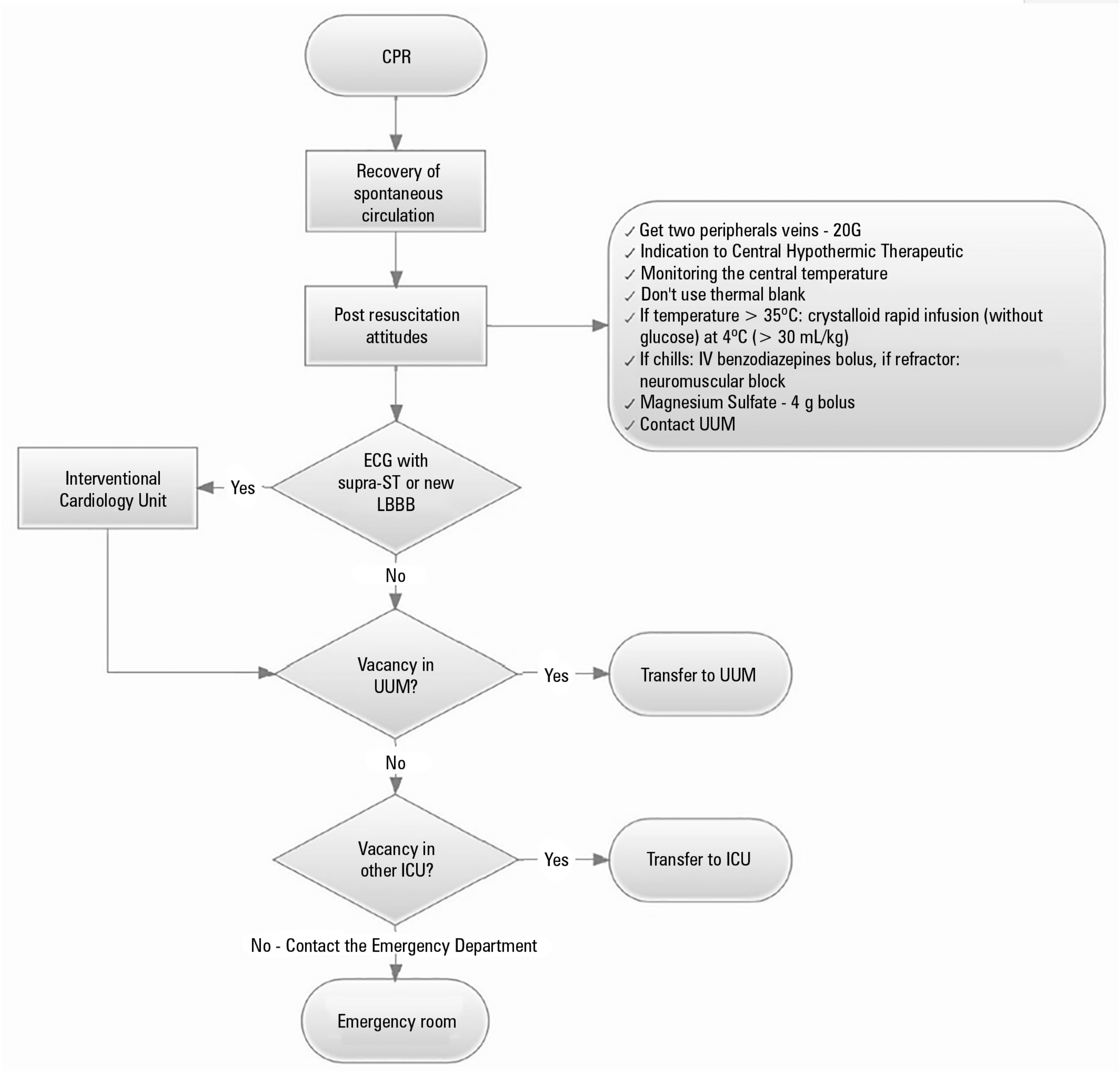

Figure 1S - Out-of-hospital patient guidance. CPR - cardiopulmonary resuscitation; ECG - electrocardiogram; LBBB - left bundle branch block; UUM - unidade de urgência médica (intensive care unit); ICU - intensive care unit.

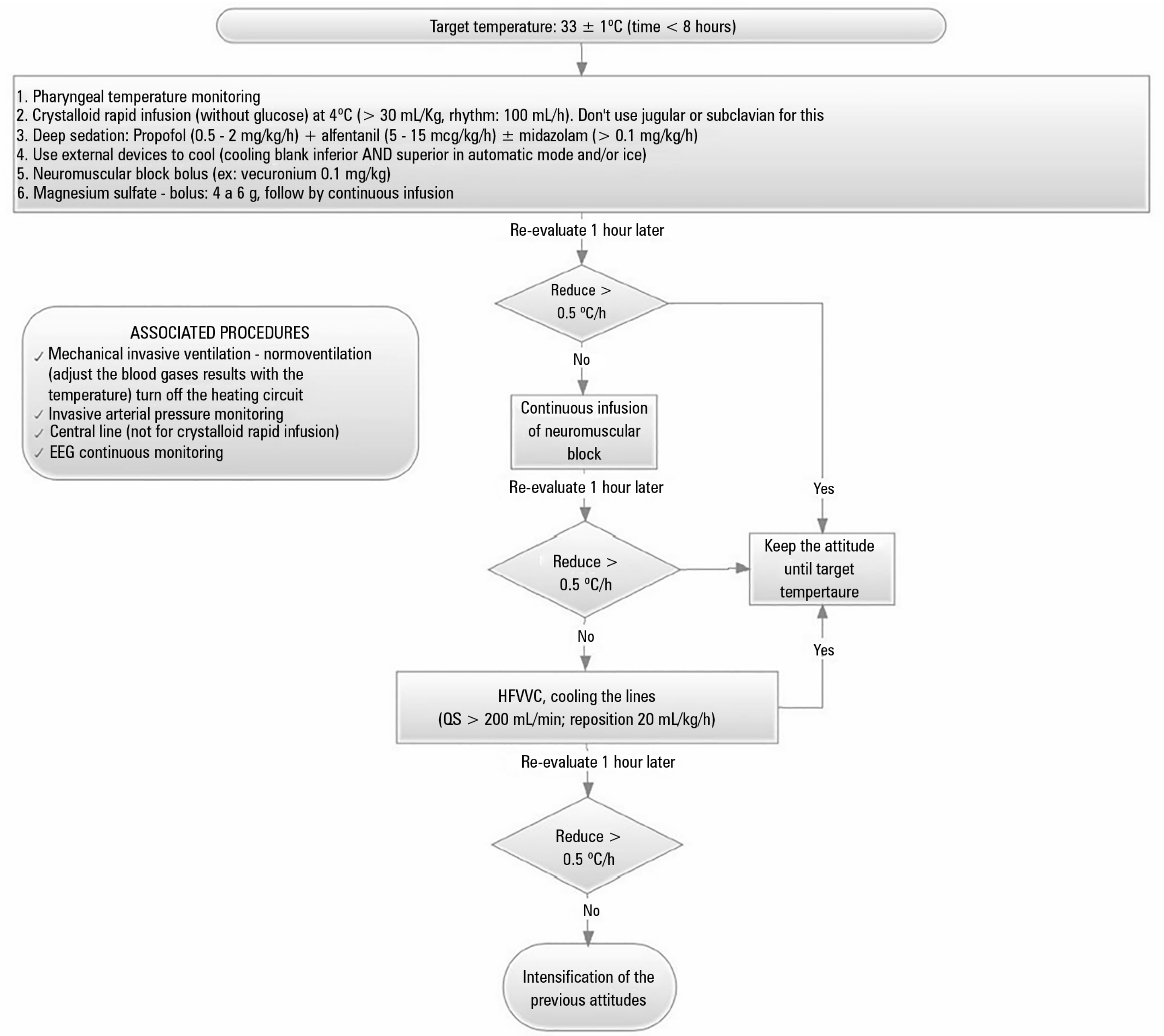

Figure 2S - Hypothermia protocol. HFVVC - continuous veno-venous hemofiltration; QS - blood debt; EEG - electroencephalogram.
